# Supplementary material for: Extended-depth of field random illumination microscopy, EDF-RIM, provides super-resolved projective imaging
Source: Light Sci Appl. 2024 Oct 10;13:285. doi: 10.1038/s41377-024-01612-0 (PMC11479626; doi:10.1038/s41377-024-01612-0)
Supplement: Supplementary file 1 — Supplementary information [file 41377_2024_1612_MOESM1_ESM.pdf]

Supplementary information for: Extended-depth of field random illumination microscopy, EDF-RIM, provides super-resolved projective imaging

Lorry Mazzella[1,+], Thomas Mangeat[2,+], Guillaume Giroussens[1], Benoit Rogez[1], Hao Li[3], Justine Creff[3], Mehdi Saadaoui[4], Carla Martins[1], Ronan Bouzignac[3], Simon Labouesse[2], Jérome Idier[5], Frédéric Galland[1], Marc Allain[1], Anne Sentenac[1,*], and Loïc LeGoff[1,*]

[1]Aix Marseille Univ, CNRS, Centrale Marseille, Institut Fresnel UMR7249, Turing Center for Living Systems, Marseille, France
[2]LITC Core Facility, Centre de Biologie Integrative (CBI), CNRS, Université de Toulouse,UT3, Toulouse, France
[3]MCD, Centre de Biologie Intégrative (CBI),CNRS,Université de Toulouse, UT3, Toulouse, France
[4]Aix Marseille Univ, CNRS, IBDM UMR7288, Turing Centre for Living systems, Marseille, France
[5]LS2N, CNRS UMR 6004, F44321 Nantes Cedex 3, France
[+]These authors contributed equally to this work
[*]Authors for correspondence : anne.sentenac@fresnel.fr, loic.le-goff@univ-amu.fr

List of Supplementary material

Appendix A: Detailed theory of EDF-RIM
Appendix B: Simulations
Supplementary Figure S1: Simulations
Supplementary Figure S2: Fourier spectra analysis
Supplementary Figure S3: Cytokinesis quantification in the *Drosophila* embryo
Supplementary Figure S4: Effect of z-sampling on the quality of surface reconstruction
Supplementary Figure S5: EDF-RIM using a stair-step phase mask
Supplementary Movie caption SM1
Supplementary Movie caption SM2
Supplementary Movie caption SM3
Supplementary Movie caption SM4
References for the Supplementary information

Appendix A: Detailed theory of EDF-RIM

Model for extend depth image formation

We write $\boldsymbol{r} := (\boldsymbol{r}_\perp, z) \in \mathbb{R}^3$ as a spatial coordinate in 3D, with $\boldsymbol{r}_\perp := (x, y)$ locating a position in the (transverse) plane perpendicular to the optical axis z. Additionally, if f is a function defined over of $\boldsymbol{r}$, we note $f_\perp$ its projection along the optical axis

$$f_\perp(\boldsymbol{r}_\perp) := \int f(\boldsymbol{r})\,\mathrm{d}z. \tag{1}$$

For a given 3D speckle excitation, the EDF (2D) image corresponds to the sum of the contribution from all planes, which writes as follows:

$$I_{\mathrm{EDF}}(\boldsymbol{r}_\perp) = \int \left(\iint \rho(\boldsymbol{r}'_\perp, z') S(\boldsymbol{r}'_\perp, z') h(\boldsymbol{r}_\perp - \boldsymbol{r}'_\perp, z - z')\,\mathrm{d}\boldsymbol{r}'_\perp \mathrm{d}z' \right) \mathrm{d}z \tag{2}$$

with h is the 3D PSF of the microscope, S the speckled illumination and ρ the fluorescence density (which is the product between the fluorophore concentration and their brightness). In the above equation, if we integrate along z first, we get

$$I_{\mathrm{EDF}}(\boldsymbol{r}_\perp) = \iint \rho(\boldsymbol{r}'_\perp, z') S(\boldsymbol{r}'_\perp, z') h_\perp(\boldsymbol{r}_\perp - \boldsymbol{r}'_\perp)\,\mathrm{d}\boldsymbol{r}'_\perp \mathrm{d}z' \tag{3}$$

where we used the fact that $\int h(\boldsymbol{r}_\perp, z - z')\mathrm{d}z = \int h(\boldsymbol{r}_\perp, z)\mathrm{d}z = h_\perp(\boldsymbol{r}_\perp)$. Let us assume further that speckles are columnar functions. In such an instance, S is invariant along z and we write $S(\boldsymbol{r}_\perp, z) = S_{\mathrm{B}}(\boldsymbol{r}_\perp)$, $\forall z$. The relation above has the following specific form

$$I_{\mathrm{B}}(\boldsymbol{r}_\perp) = \iint \rho(\boldsymbol{r}'_\perp, z') S_{\mathrm{B}}(\boldsymbol{r}'_\perp) h_\perp(\boldsymbol{r}_\perp - \boldsymbol{r}'_\perp)\,\mathrm{d}\boldsymbol{r}'_\perp \mathrm{d}z' \tag{4}$$

and if we finally integrate along z', we obtain a simple convolution model that involves projected quantities only

$$I_{\mathrm{B}}(\boldsymbol{r}_\perp) = \int \rho_\perp(\boldsymbol{r}'_\perp) S_{\mathrm{B}}(\boldsymbol{r}'_\perp) h_\perp(\boldsymbol{r}_\perp - \boldsymbol{r}'_\perp)\,\mathrm{d}\boldsymbol{r}'_\perp. \tag{5}$$

In summary, under the assumption of a Bessel illumination, the model for the microscope image is analogous to the one of a standard widefield microscope, but it involves the extended depth PSF, $h_\perp$ and the projection of the object $\rho_\perp$.

Theoretical expression of the EDF variance

We derive now the theoretical variance of a EDF-RIM experiment. We start first by making no specific assumption about the illumination function $S(\boldsymbol{r}_\perp, z)$. More specifically, we write from Eq.3:

$$\begin{aligned}
\sigma^2_{\mathrm{EDF}}(\boldsymbol{r}_\perp) &:= \langle I^2_{\mathrm{EDF}}(\boldsymbol{r}_\perp)\rangle - \langle I_{\mathrm{EDF}}(\boldsymbol{r}_\perp)\rangle^2 \\
&= \iint \left\langle \int \rho(\boldsymbol{r}'_\perp, z') S(\boldsymbol{r}'_\perp, z')\,\mathrm{d}z' \int \rho(\boldsymbol{r}''_\perp, z'') S(\boldsymbol{r}''_\perp, z'')\,\mathrm{d}z'' \right\rangle h_\perp(\boldsymbol{r}_\perp - \boldsymbol{r}'_\perp) h_\perp(\boldsymbol{r}_\perp - \boldsymbol{r}''_\perp)\,\mathrm{d}\boldsymbol{r}'_\perp\,\mathrm{d}\boldsymbol{r}''_\perp \\
&\quad - \int \left\langle \int \rho(\boldsymbol{r}'_\perp, z') S(\boldsymbol{r}'_\perp, z')\,\mathrm{d}z' \right\rangle h_\perp(\boldsymbol{r}_\perp - \boldsymbol{r}'_\perp)\,\mathrm{d}\boldsymbol{r}'_\perp \int \left\langle \int \rho(\boldsymbol{r}''_\perp, z'') S(\boldsymbol{r}''_\perp, z'')\,\mathrm{d}z'' \right\rangle h_\perp(\boldsymbol{r}_\perp - \boldsymbol{r}''_\perp)\,\mathrm{d}\boldsymbol{r}''_\perp
\end{aligned} \tag{6}$$

Assuming speckles that are invariant along z, we can write $S(\boldsymbol{r}_\perp, z) = S_{\mathrm{B}}(\boldsymbol{r}_\perp)$ which allows the following simplification

$$\int \rho(\boldsymbol{r}_\perp, z) S(\boldsymbol{r}_\perp, z)\,\mathrm{d}z = \rho_\perp(\boldsymbol{r}_\perp) S_{\mathrm{B}}(\boldsymbol{r}_\perp) \tag{7}$$

hence leading to the following approximation for the EDF-RIM variance

$$\sigma^2_{\mathrm{EDF}}(\boldsymbol{r}_\perp) \approx \sigma^2_{\mathrm{B}}(\boldsymbol{r}_\perp) := \iint \rho_\perp(\boldsymbol{r}'_\perp) \rho_\perp(\boldsymbol{r}''_\perp) \Gamma_{\mathrm{B}}(\boldsymbol{r}''_\perp - \boldsymbol{r}'_\perp) h_\perp(\boldsymbol{r}_\perp - \boldsymbol{r}'_\perp) h_\perp(\boldsymbol{r}_\perp - \boldsymbol{r}''_\perp)\,\mathrm{d}\boldsymbol{r}'_\perp\,\mathrm{d}\boldsymbol{r}''_\perp \tag{8}$$

where we have introduced the 2D auto-covariance of the columnar speckle $\Gamma_{\mathrm{B}}(\boldsymbol{r}''_\perp - \boldsymbol{r}'_\perp) = \langle S_{\mathrm{B}}(\boldsymbol{r}'_\perp) S_{\mathrm{B}}(\boldsymbol{r}''_\perp)\rangle - \langle S_{\mathrm{B}}(\boldsymbol{r}'_\perp)\rangle \langle S_{\mathrm{B}}(\boldsymbol{r}''_\perp)\rangle$. The right-hand side in Eq. 8 has the structure of the variance expression in RIM given by Eq. 3 in the main text. It should be noted, however, that the classical result $h_\perp = \Gamma_{\mathrm{B}}(\boldsymbol{r}_\perp)$ will not be verified, unless we modify the observation PSF by putting a ring in the pupil (Fourier plane).

EDF-RIM variance with standard speckles: the case of a spatially smooth fluorescence surface

Bessel-speckles are rarely used as an illumination function. Here, we investigate under what conditions EDF-RIM can be performed with conventional 3D speckles. We thus return to the general case of an illumination speckle that is not invariant along z. Using Eq. 6, the EDF-RIM variance reads

$$\sigma^2_{\mathrm{EDF}}(\boldsymbol{r}_\perp) = \iint h_\perp(\boldsymbol{r}_\perp - \boldsymbol{r}'_\perp) h_\perp(\boldsymbol{r}_\perp - \boldsymbol{r}''_\perp) \iint \rho(\boldsymbol{r}'_\perp, z')\rho(\boldsymbol{r}''_\perp, z'')\Gamma_S(\boldsymbol{r}''_\perp - \boldsymbol{r}'_\perp, z'' - z')\, \mathrm{d}z'\, \mathrm{d}z''\, \mathrm{d}\boldsymbol{r}'_\perp\, \mathrm{d}\boldsymbol{r}''_\perp \tag{9}$$

where we have introduced the 3D auto-covariance of the speckle $\Gamma_S(\boldsymbol{r}''_\perp - \boldsymbol{r}'_\perp, z'' - z') = \langle S(\boldsymbol{r}'_\perp, z')S(\boldsymbol{r}''_\perp, z'')\rangle - \langle S(\boldsymbol{r}'_\perp, z')\rangle\langle S(\boldsymbol{r}''_\perp, z'')\rangle$. Let us assume now that the sample is such that the fluorophores are distributed along a surface denoted $Z(\boldsymbol{r}_\perp)$:

$$\rho(\boldsymbol{r}_\perp, z) = \rho(\boldsymbol{r}_\perp)\delta(z - Z(\boldsymbol{r}_\perp)) \tag{10}$$

where δ is the Dirac distribution. Under this assumption, Eq. 9 simplifies into

$$\sigma^2_{\mathrm{EDF}}(\boldsymbol{r}_\perp) = \iint \rho(\boldsymbol{r}'_\perp)\rho(\boldsymbol{r}''_\perp)\Gamma_S\left(\boldsymbol{r}''_\perp - \boldsymbol{r}'_\perp, Z(\boldsymbol{r}''_\perp) - Z(\boldsymbol{r}'_\perp)\right) h_\perp(\boldsymbol{r}_\perp - \boldsymbol{r}'_\perp) h_\perp(\boldsymbol{r}_\perp - \boldsymbol{r}''_\perp)\, \mathrm{d}\boldsymbol{r}'_\perp\, \mathrm{d}\boldsymbol{r}''_\perp$$

This expression is similar to the standard 2D RIM expression of the variance (Eq. 3 in the main text) except that it involves $\Gamma_S\left(\boldsymbol{r}''_\perp - \boldsymbol{r}'_\perp, Z(\boldsymbol{r}''_\perp) - Z(\boldsymbol{r}'_\perp)\right)$ rather than $\Gamma_S\left(\boldsymbol{r}''_\perp - \boldsymbol{r}'_\perp, 0\right)$. To further simplify the speckle auto-covariance into a 2D expression, we hypothesize that the objects distribute on a smooth topography. This translates mathematically into the following assumption for Γ_S:

- $||\boldsymbol{r}''_\perp - \boldsymbol{r}'_\perp|| < \frac{\lambda}{2\mathrm{NA}} \implies Z(\boldsymbol{r}''_\perp) \approx Z(\boldsymbol{r}'_\perp) \implies \Gamma_S(\boldsymbol{r}''_\perp - \boldsymbol{r}'_\perp, Z(\boldsymbol{r}''_\perp) - Z(\boldsymbol{r}'_\perp)) \approx \Gamma_S(\boldsymbol{r}''_\perp - \boldsymbol{r}'_\perp, 0)$
- $||\boldsymbol{r}''_\perp - \boldsymbol{r}'_\perp|| > \frac{\lambda}{2\mathrm{NA}} \implies \Gamma_S(\boldsymbol{r}''_\perp - \boldsymbol{r}'_\perp, z'' - z') \approx 0$

Two assumptions have been made. The first one states that the surface does not vary much in z over lateral distances of the order of $\frac{\lambda}{2NA}$ (smoothness hypothesis). The second one neglects long-range correlations in the illumination, which is ensured because speckle decorrelates on length-scales similar to the lateral extent of the PSF, $\frac{\lambda}{2NA}$[1]. Following these assumptions, the expression of the variance of the intensity is simplified: it involves the 2D PSF $h_\perp$ and the 2D auto-covariance function $\Gamma_{\mathrm{EDF}}(\boldsymbol{r}_\perp) := \Gamma_S(\boldsymbol{r}_\perp, 0)$:

$$\sigma^2_{\mathrm{EDF}}(\boldsymbol{r}_\perp) \approx \iint \rho(\boldsymbol{r}'_\perp)\rho(\boldsymbol{r}''_\perp)\Gamma_{\mathrm{EDF}}(\boldsymbol{r}''_\perp - \boldsymbol{r}'_\perp) h_\perp(\boldsymbol{r}_\perp - \boldsymbol{r}'_\perp) h_\perp(\boldsymbol{r}_\perp - \boldsymbol{r}''_\perp)\, \mathrm{d}\boldsymbol{r}'_\perp\, \mathrm{d}\boldsymbol{r}''_\perp \tag{11}$$

To conclude, our study has shown that the variance of the extended depth RIM images can be simplified into the classical RIM 2D expression (Eq. 3 in the main text) for any type of sample, by using columnar speckles (see Eq. 8), and for surface and smoothed samples, when using regular 3D speckles (see Eq. 11).

In the following section, we demonstrate an experimental implementation of EDF-RIM. We then investigate the performance of EDF-RIM and the limits of its numerical approach through simulations.

Appendix B: Simulations

We investigate the realms of application of EDF-RIM through simulations. More specifically, we examine the implications of the EDF variance expression in the case of the 3D speckle (Eq. 9) only approximately conforming to the canonical 2D-RIM expression (Eq. 3 of the main text). In the simulations, the imaged object is characterized by its star-shaped lateral distribution $\rho(r)$, as shown in Fig. S1.A (left). We investigate three distinct topographies for the 3D object, as depicted in Fig. S1.A (right). These include a flat structure, a smoothly varying surface in shape of a cone, and a random distribution of z-position. The flat object serves as a reference for which our model is correct, with no approximation. The smoothly varying surface satisfies the approximation conditions for the EDF variance to align with the canonical 2D-RIM expression (Eq. 3 in the main text), whereas the random configuration does not.

From a numerical standpoint, the inversion requires the deconvolution of the variance of the prefiltered images ($\eta = 10^{-6}$) images using a Tikhonov regularized inverse filter[2]. The effectiveness of this inverse filter depends on its regularization parameter. When this parameter is too high, it can result in image blurring and prevent super-resolution. Conversely, setting it too low may lead to the amplification of noise and the appearance of artefacts. We compared the inversions for 2 different regularization parameters -one large (10^{-5}) and one low (10^{-10} respectively), and for the 3 aforementioned 3D objects, the idea being that an inaccurate model generally induces more artifacts for small regularization parameters.

We first examine the scenario of a 3D propagating speckle, which aligns with our experimental implementation. In Fig. S1.B, we present the reconstructed images for the three topographies, with both large ($\mu = 10^{-5}$) and small ($\mu = 10^{-10}$) Tikhonov regularization parameters. While all three results appear satisfactory at $\mu = 10^{-5}$, this setting is over-regularized, leading to a suboptimal resolution. At $\mu = 10^{-10}$, only the smooth surface compares well with the 2D case, while reconstruction of the random z-position is thwarted by amplified noise. Thus, it stands that conditions that permit the use of an exact or at least approximate expression for the image variance are a prerequisite for EDF-RIM.

As outlined in Appendix A, the EDF-variance expression can be transformed into a 2D-RIM expression for all types of samples when using a speckle that remains invariant along the optical axis. This condition is achieved experimentally by blocking light outside an annulus in the pupil plane (Bessel speckle, Fig. S1.C, right). We simulated these Bessel speckles by modifying the binary mask in the pupil plane which is no longer a disk but an annulus. This annulus filters out all frequencies smaller than $0.95\frac{2NA}{\lambda}$ and bigger than $\frac{2NA}{\lambda}$. The generated speckle are effectively invariant along the optical axis (Fig. S1.C, right panels).

Simulating EDF-RIM with these Bessel speckles, we were able to achieve high-resolution images for all samples, including the random z configuration, for every value of the regularization parameter tested (Fig. S1.B, lower panels). This is consistent with our prediction that with columnar speckles, the reconstruction does not rely on the sparsity hypothesis.

In conclusion, our simulations confirm that EDF-RIM is a feasible imaging method, and support the limits outlined in the theory section. Specifically, we have demonstrated that 3D-speckles can be used to image sparse samples along the optical axis, such as surfaces, and Bessel speckles can used for arbitrary samples.

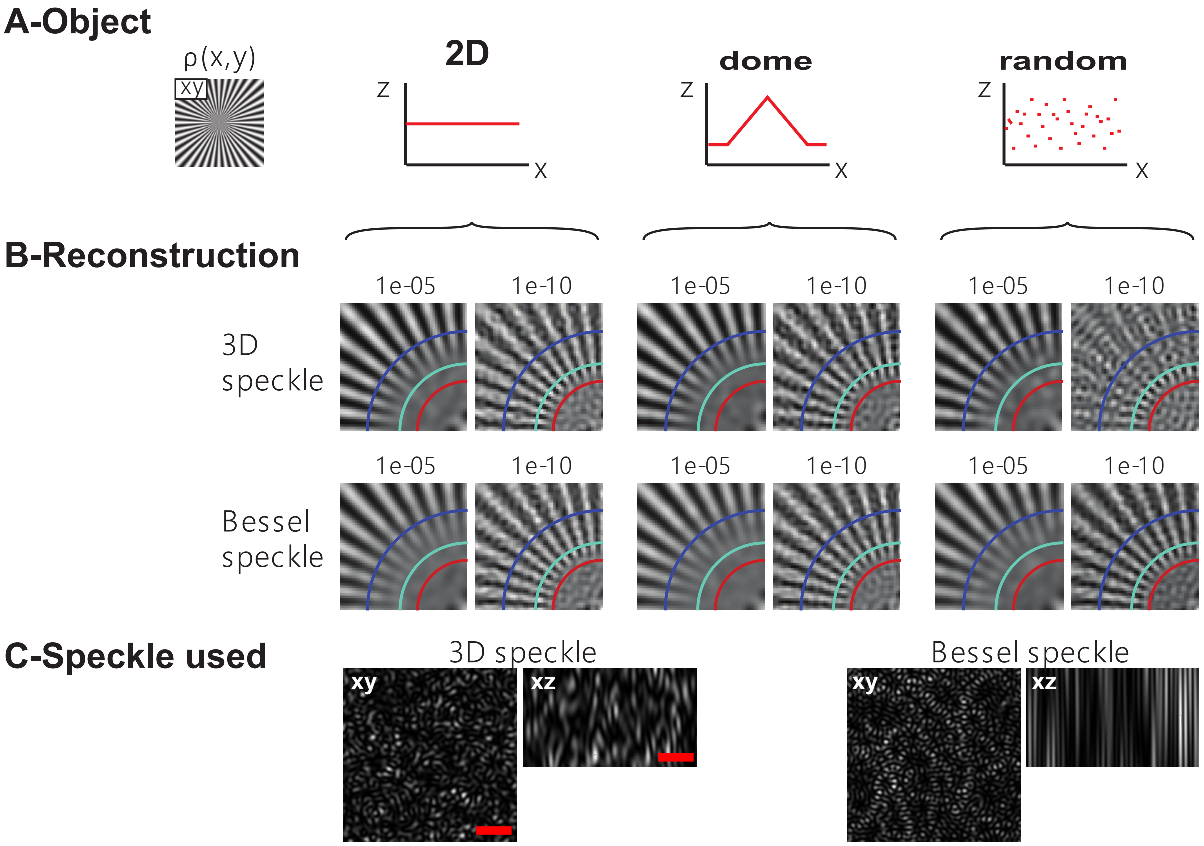

Figure S1. Simulations. A) Simulated object: the lateral distribution $\rho(r)$ is depicted on the left, while three topographies are studied – a flat structure (left), a dome (center) and a random topography (right). B) Simulated reconstructions for the 3 topographies studied, with 2 different illumination-patterns (see below). For each condition, we consider the reconstruction using a large and a small regularization parameter in the inversion procedure (10^{-5} and 10^{-10} respectively). Images include guidelines at $\frac{\lambda}{2NA}$ (blue circle), $\frac{\lambda}{2.96NA}$ (cyan circle) and $\frac{\lambda}{4NA}$ (red circle). C) The three dimensional illumination patterns used in the simulation (scale bar: 2 μm). The 3D-Speckle is obtained numerically by simulating the propagation of an electromagnetic field with uniform amplitude and random phase distribution in the back focal plane of the objective. The Bessel speckle is obtained by replacing the homogeneous amplitude in the back focal plane of the lens with an annulus illumination.

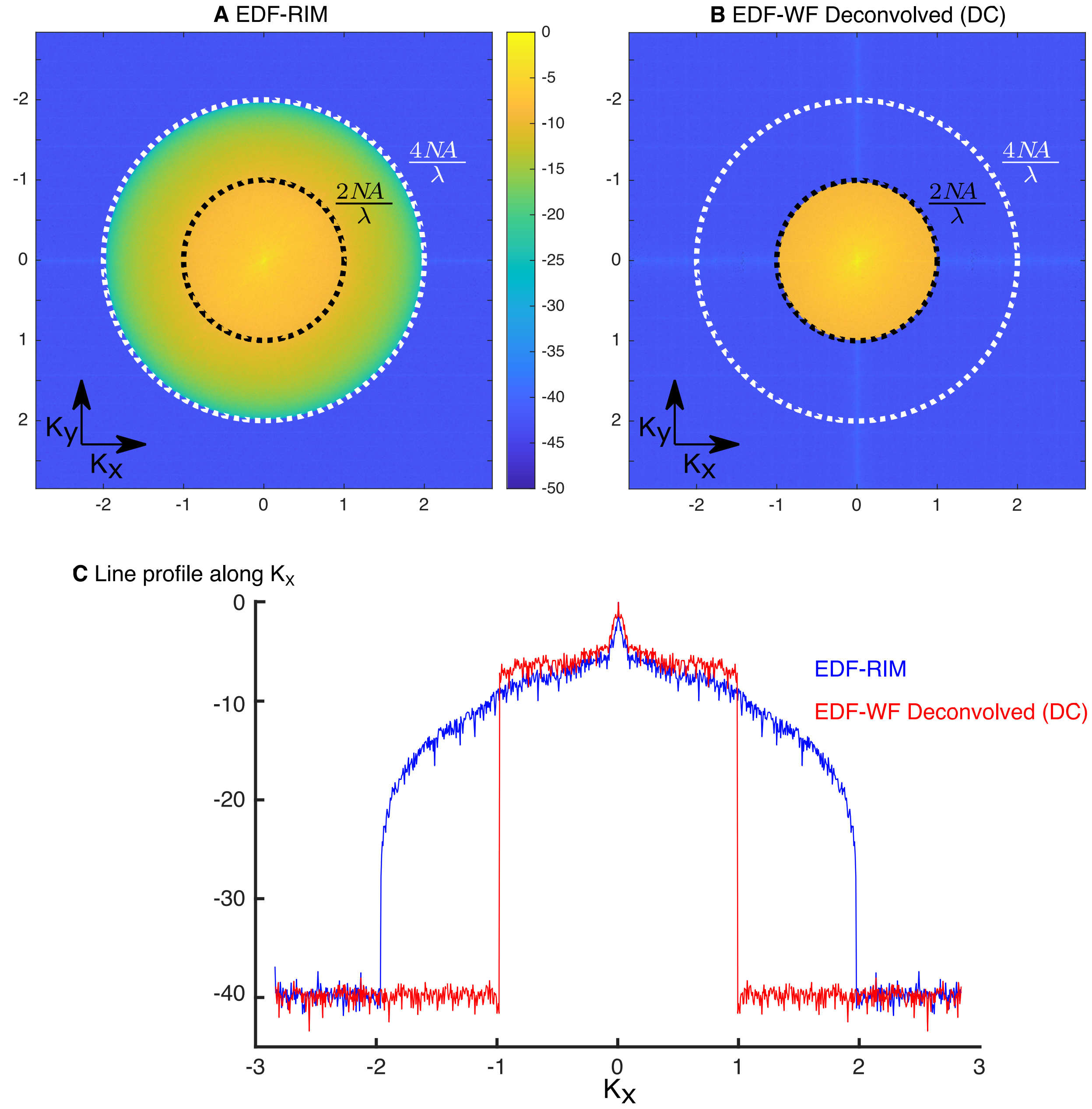

Figure S2. Fourier spectral analysis (A,B) correspond to the Fourier spectra in log scale of Fig. 2A,C. Spatial frequencies are normalized by the OTF cut-off frequency $f_c = \frac{2NA}{\lambda}$ with $NA = 1.21$ and $\lambda = 530$ nm. The black and white circles indicate the frequencies f_c and $2f_c$, respectively. (C) Line profile along K_x for $K_y = 0$ of the EDF-RIM Fourier spectra (blue curve) and deconvolved EDF-widefield (red curve). The Fourier spectrum of deconvolved EDF-widefield is limited to f_c while the Fourier spectrum of EDF-RIM is limited to $2f_c$. There is an intensity decay in the Fourier spectrum of EDF-RIM at roughly $1.7f_c$. By using smaller regularization parameters, this value could be improved to be closer to $2f_c$, but would generate more artefacts.

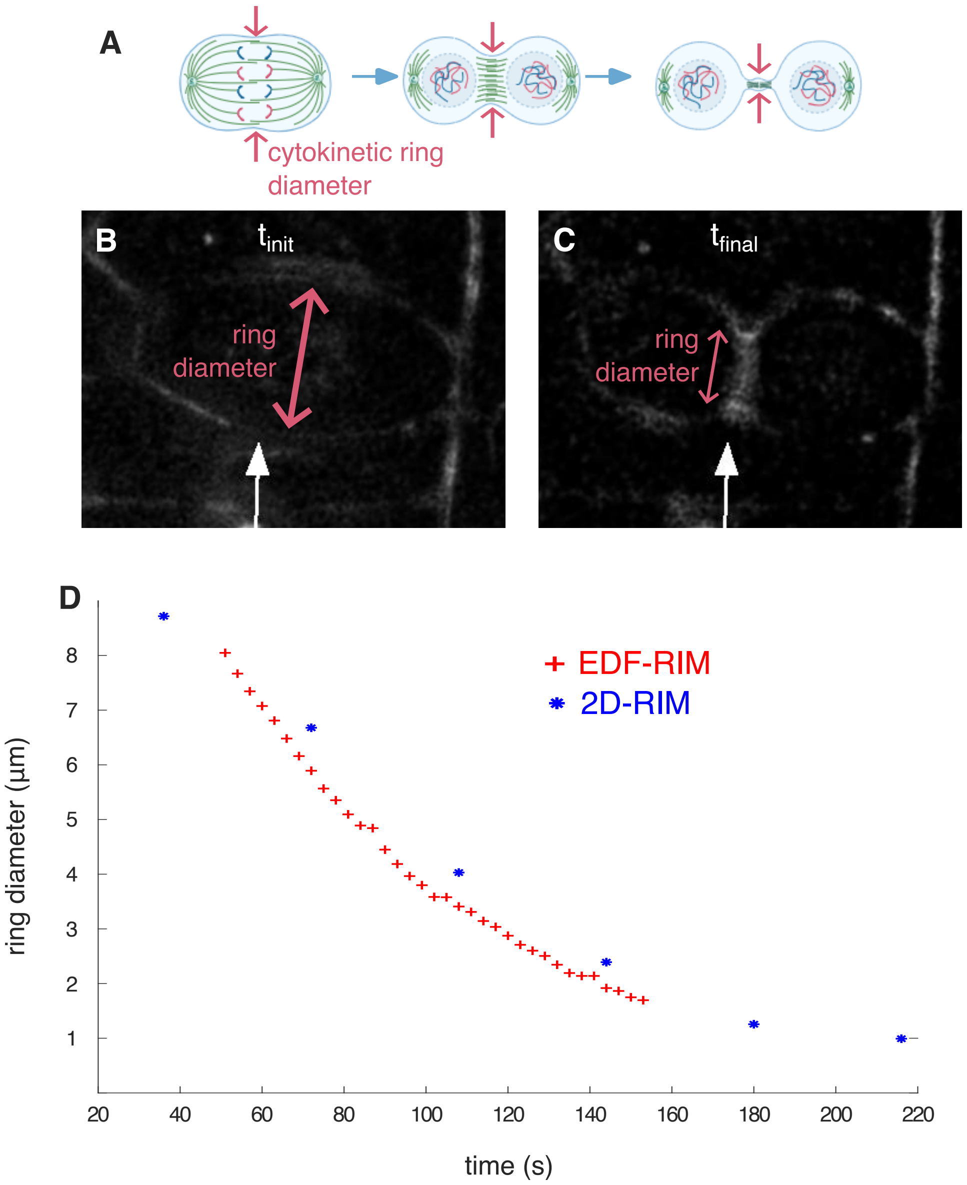

Figure S3. Cytokinesis quantification in the *Drosophila* embryo. Quantification of cytokinetic ring contraction taken from Supplementary movies 1 . A) Schematic of a dividing cell showing the cytokinetic ring, measured in this figure. B,C) Snapshots from Supplementary movie 1, in which the white arrows point to dividing cells. D) Ring contraction as measured from Supplementary movie 1 using EDF-RIM and Supplementary movie 2 using 2D-RIM. Each point corresponds to one time point from the movie. The superior temporal resolution of EDF-RIM is reflected in the more than 10-fold increased density of measurement points. The reduced temporal resolution in 2D-RIM results in jitter, than makes cell divisions hard to follow (white arrow in Supplementary movie 2).

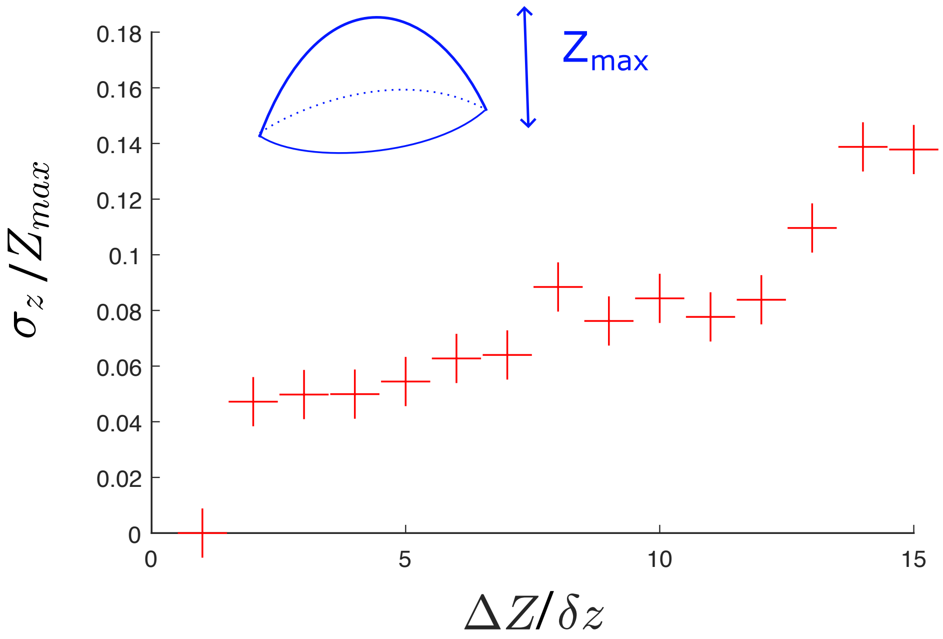

Figure S4. Effect of z-sampling on the quality of surface reconstruction. To estimate the dependence of the surface reconstruction on z-sampling, we estimate the surface using increasing Z intervals $\Delta Z = n.\delta z$, where $\delta = 177nm$ is the minimal interval used, and n is an integer. The error with respect to the best estimation (n=1), is quantified as the root mean squared normalized by the height of the tissue (Z_{max}, see inset on top left of image). The z-dependence is quite low up to $n = 12$ corresponding to $\Delta Z \simeq 2.1\mu m$.

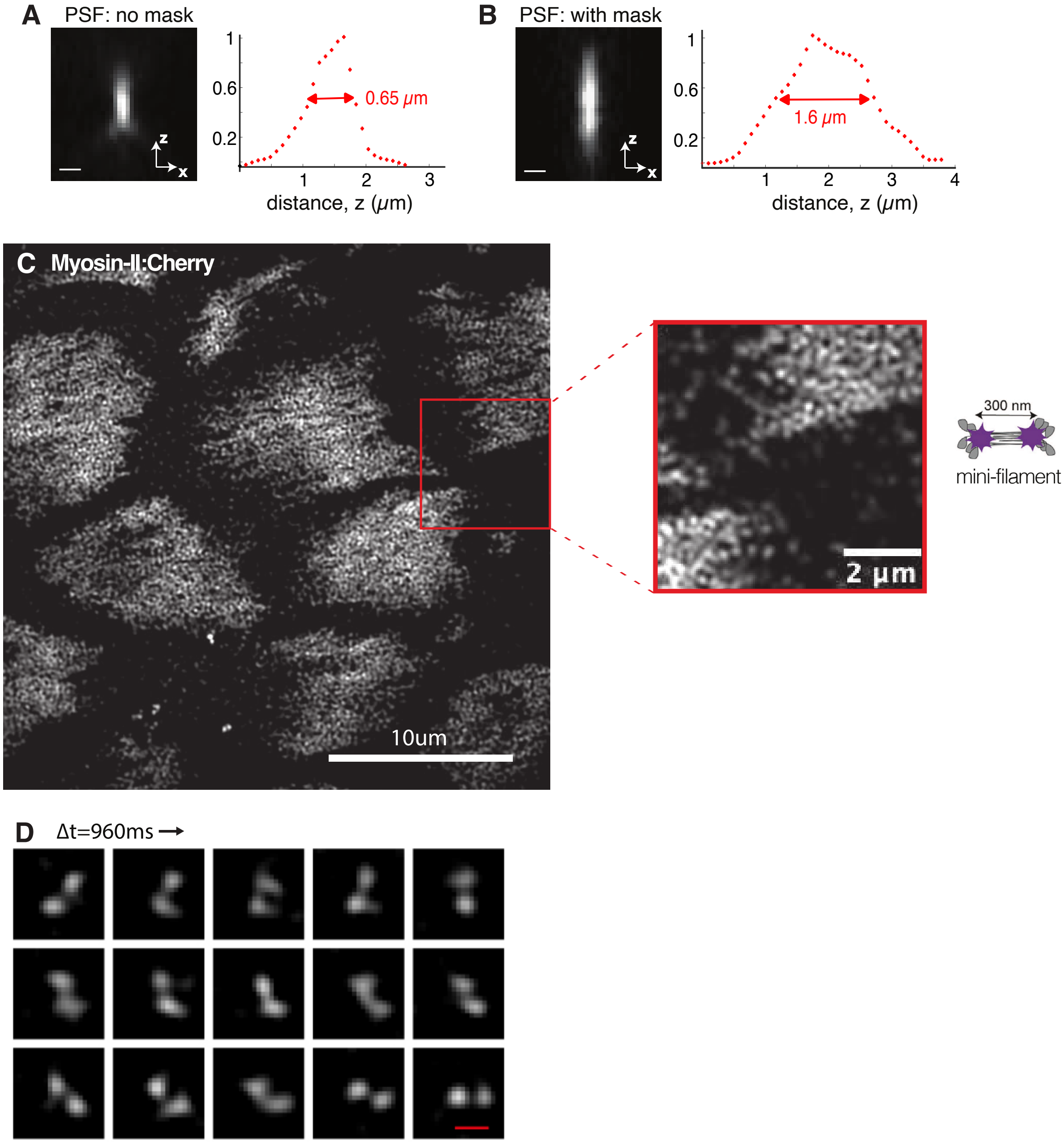

Figure S5. **EDF-RIM using a stair-step phase mask.** The ETL was replaced with a stair-step phase mask[3]. (A,B) The net result is an extension of the axial PSF from 0.65μm to 1.60μm. (C) Application to MyoII imaging in a *Drosophila* egg chamber. The schematic on the right shows the molecular structure of a typical mini-filament. The anti-polar arrangement leads to a fluorescent structure consisting of two fluorescent points, rigidly linked and spaced by approximately 300nm. (D) Close-up on sequences, imaged with a 960 ms interval, showing the rotation of an individual Myosin-II mini-filament, a typical behavior that was described in[4]. Scales bars=300 nm when not specified.

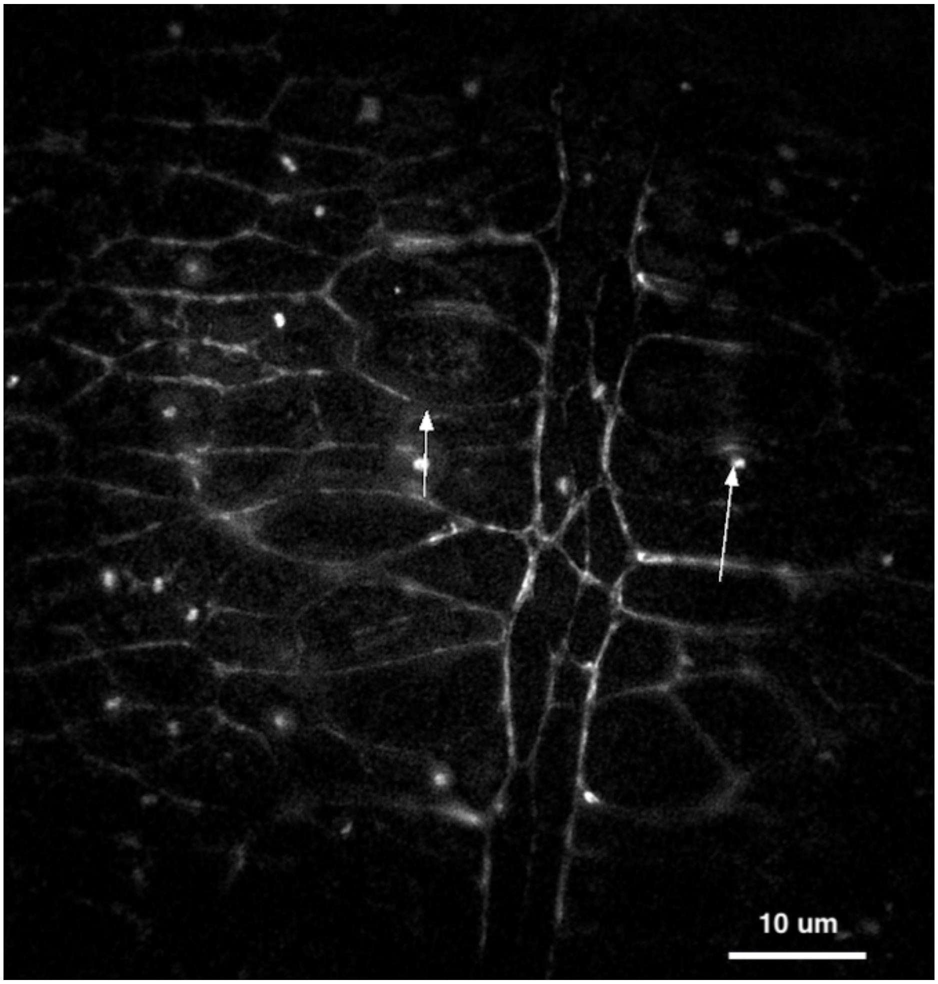

Figure SM1. Supplementary movie 1 caption. Myosin:TagRFP imaging in the Drosophila embryo with EDF-RIM, in link with Fig. S3. White arrows point to closing cytokinetic rings. The temporal resolution is improved compared to Supplementary Movie 2.

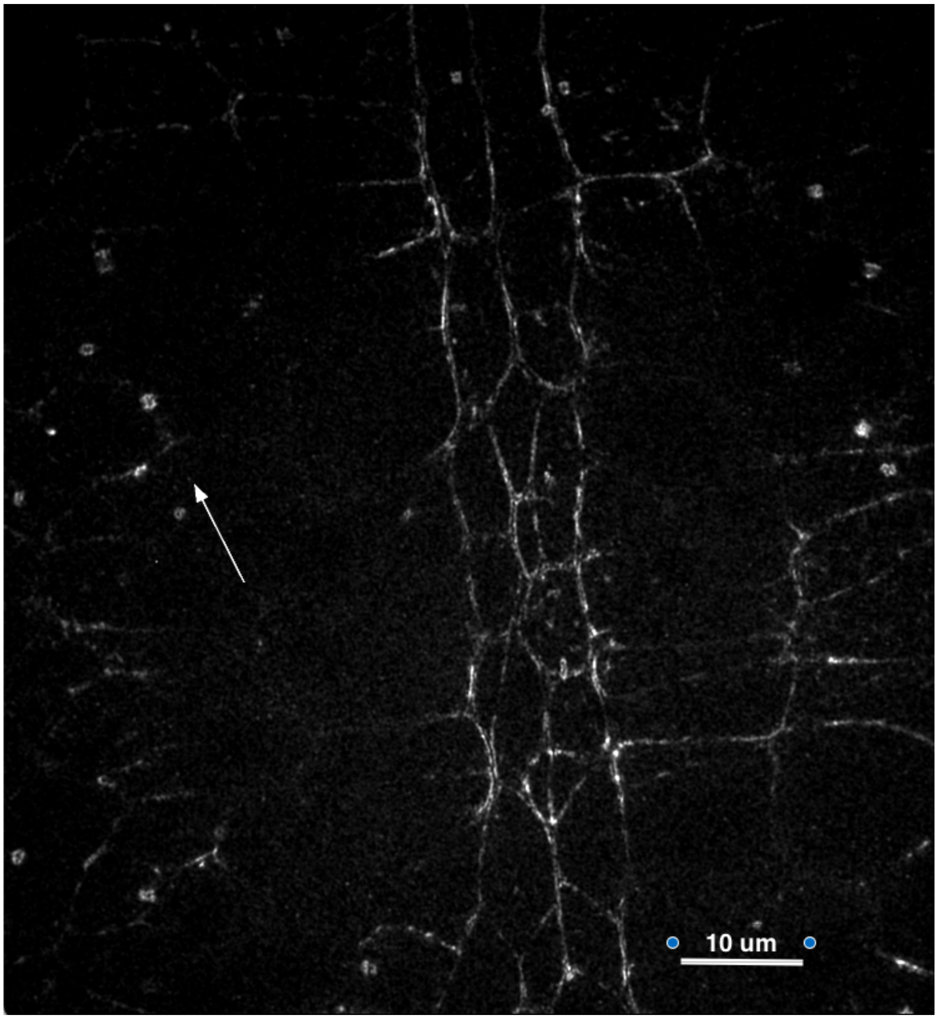

Figure SM2. **Supplementary movie 2 caption.** Myosin:TagRFP imaging in the Drosophila embryo with 2D-RIM in link with Fig. S3. Multiple planes are acquired sequentially, and summed for projection. White arrows point to closing cytokinetic rings. The temporal resolution is decreased compared to Supplementary Movie 1.

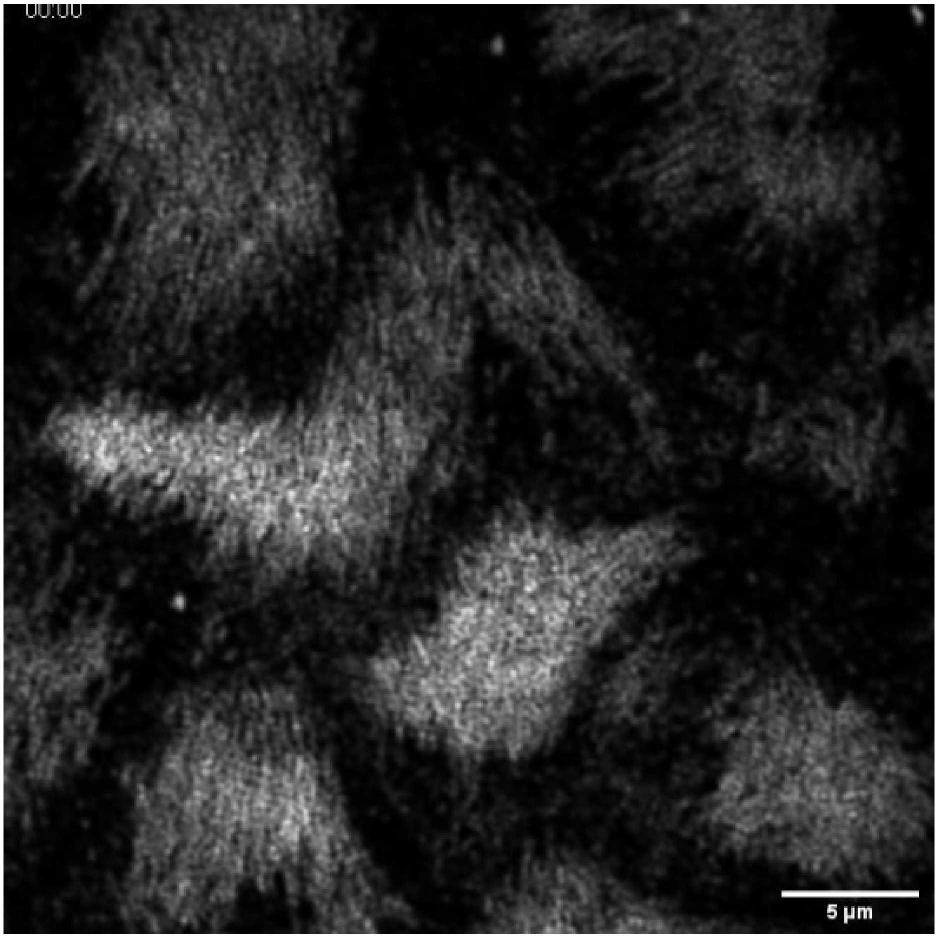

Figure SM3. Supplementary movie 3 caption. Myosin-II:GFP in the basal surface of follicle cells from a Drosophila egg chamber in link with Fig. S5C.

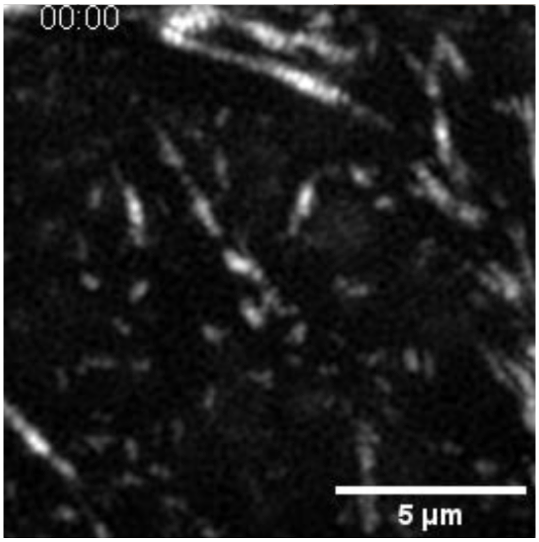

Figure SM4. Supplementary movie 4 caption. Myosin-II:GFP in the basal surface of follicle cells from a Drosophila egg chamber in link with Fig. S5D

References

1. Goodman, J. W. Some fundamental properties of speckle. *JOSA* **66**, 1145–1150 (1976).
2. Mangeat, T. *et al.* Super-resolved live-cell imaging using random illumination microscopy. *Cell Reports Methods* **1**, 100009 (2021).
3. Abrahamsson, S., Usawa, S. & Gustafsson, M. A new approach to extended focus for high-speed high-resolution biological microscopy. *Three-Dimensional Multidimens. Microsc. Image Acquis. Process. XIII* **6090**, 128–135 (2006).
4. Hu, S. *et al.* Long-range self-organization of cytoskeletal myosin ii filament stacks. *Nat. cell biology* **19**, 133–141 (2017).
